# Exploring Older Adults’ Perspectives and Acceptance of AI-Driven Health Technologies: Qualitative Study

**DOI:** 10.2196/66778

**Published:** 2025-02-12

**Authors:** Arkers Kwan Ching Wong, Jessica Hiu Toon Lee, Yue Zhao, Qi Lu, Shulan Yang, Vivian Chi Ching Hui

**Affiliations:** 1 School of Nursing, The Hong Kong Polytechnic University, 1 Yuk Choi Rd, Hung Hom, Kowloon, Hong Kong, China (Hong Kong), 852 34003805; 2 School of Nursing, Tianjin Medical University, Tianjin, China; 3 Nursing Department, Zhejiang Hospital, Hangzhou, China

**Keywords:** artificial intelligence–based health technologies, health technology, AI-based health technology, machine learning, ML, artificial intelligence, AI, algorithm, model, analytics, perceptions, acceptability, gerontology, geriatrics, older adult, elderly, older person, older people, aging, mobile phone

## Abstract

**Background:**

Artificial intelligence (AI) is increasingly being applied in various health care services due to its enhanced efficiency and accuracy. As the population ages, AI-based health technologies could be a potent tool in older adults’ health care to address growing, complex, and challenging health needs. This study aimed to investigate perspectives on and acceptability of the use of AI-led health technologies among older adults and the potential challenges that they face in adopting them. The findings from this inquiry could inform the designing of more acceptable and user-friendly AI-based health technologies.

**Objective:**

The objectives of the study were (1) to investigate the attitudes and perceptions of older adults toward the use of AI-based health technologies; (2) to identify potential facilitators, barriers, and challenges influencing older adults’ preferences toward AI-based health technologies; and (3) to inform strategies that can promote and facilitate the use of AI-based health technologies among older adults.

**Methods:**

This study adopted a qualitative descriptive design. A total of 27 community-dwelling older adults were recruited from a local community center. Three sessions of semistructured interviews were conducted, each lasting 1 hour. The sessions covered five key areas: (1) general impressions of AI-based health technologies; (2) previous experiences with AI-based health technologies; (3) perceptions and attitudes toward AI-based health technologies; (4) anticipated difficulties in using AI-based health technologies and underlying reasons; and (5) willingness, preferences, and motivations for accepting AI-based health technologies. Thematic analysis was applied for data analysis. The Theoretical Domains Framework and the Capability, Opportunity, Motivation, and Behavior (COM-B) model behavior change wheel were integrated into the analysis. Identified theoretical domains were mapped directly to the COM-B model to determine corresponding strategies for enhancing the acceptability of AI-based health technologies among older adults.

**Results:**

The analysis identified 9 of the 14 Theoretical Domains Framework domains—knowledge, skills, social influences, environmental context and resources, beliefs about capabilities, beliefs about consequences, intentions, goals, and emotion. These domains were mapped to 6 components of the COM-B model. While most participants acknowledged the potential benefits of AI-based health technologies, they emphasized the irreplaceable role of human expertise and interaction. Participants expressed concerns about the usability of AI technologies, highlighting the need for user-friendly and tailored AI solutions. Privacy concerns and the importance of robust security measures were also emphasized as critical factors affecting their willingness to adopt AI-based health technologies.

**Conclusions:**

Integrating AI as a supportive tool alongside health care providers, rather than regarding it as a replacement, was highlighted as a key strategy for promoting acceptance. Government support and clear guidelines are needed to promote ethical AI implementation in health care. These measures can improve health outcomes in the older adult population by encouraging the adoption of AI-driven health technologies.

## Introduction

### Background

Artificial intelligence (AI) refers to a computerized system that is capable of executing a wide range of tasks that typically involve human intelligence—ranging from physical tasks, cognitive functions, and problem-solving to decision-making—and can be performed without explicit instructions from humans [1]. AI can be classified into 4 main subsets—machine learning, natural language processing (NLP), physical robots, and robotic process automation. The field of AI is rapidly advancing, and AI technologies have been widely applied in different aspects of health care services, including diagnostic assistance, health screening, and imaging interpretations [2]. It has been described as a “second set of eyes” for medical practitioners [3]. Furthermore, over the past decade, the application of AI systems has increasingly centered on empowering patients to actively manage their health and boost their participation in the shared decision-making process, giving rise to diverse AI-driven products such as robots, smart assistants, virtual or augmented reality, wearable devices, and mobile apps [4]. Particularly notable is the emergence of AI-powered chatbot services, which permit patients to seek medical advice and receive triage for their conditions in a prompt and cost-effective manner [5]. With the debut of ChatGPT by OpenAI in November 2022 and the popularity it has gained, it is envisioned that AI-based conversational large language models with NLP abilities could conceivably revolutionize health care practice and education, contributing to substantial transformative shifts [6,7].

The world is currently encountering a significant expansion in the aging population, with the number of people aged 60 years or above anticipated to rise to 1.4 billion by 2030 and 2.1 billion by 2050 [8]. Conventional older adult health care has relied heavily on in-person monitoring; however, the further intensification of the shortage of health care workers could in the long run present a global challenge to the sustainability of delivering quality medical care [9]. In recent years, digital technologies, such as smart older adult health care products, which involve the deep integration of AI, have been used in older adult health care sectors [10]. Evidence has shown that AI-based technologies can play a constructive role in improving the physical and psychological well-being, quality of life, and independent living of older adults [11]. However, there is a great digital divide between the older and younger generations, with the former being the age group that is the least likely to have access to computers and the internet, due to physical obstacles such as physical disabilities and to psychological factors such as a lack confidence in using technology as well as ethical concerns [12,13].

While AI-based health technologies have rapidly evolved, and some studies have comprehensively discussed the advantages of applying them in the field of geriatrics, insufficient academic attention has been paid to acquiring a clear understanding of the attitudes, perceptions, and experiences of seniors toward these technologies [10]. This gap may implicitly influence the acceptance of, and motivation to use, these technologies in the future. In addition, while studies have primarily focused on the perceptions of medical practitioners and patients regarding AI health technologies, the perspectives of older adults—a key user group—have received limited attention [14-16]. For instance, although some research has explored older adults’ views on general AI-powered technologies, their relevance to AI health technologies remains uncertain [17]. Concerns about accuracy, reliability, and trust in AI tools compared with in-person medical advice further underscore the need for targeted investigation. This study addresses this gap by exploring older adults’ perceptions of and acceptability toward AI-based health technologies, with a specific focus on practical strategies to enhance adoption. By identifying barriers, facilitators, and preferences, the findings have clear clinical implications, offering actionable insights for health care providers, policy makers, and AI developers. These insights can inform the design of tailored, user-friendly AI tools and guide their ethical implementation in health care, ultimately improving older adults’ health outcomes.

### Theoretical Framework

The interview guide for this study was developed using the Theoretical Domains Framework (TDF) and the Capability, Opportunity, Motivation, and Behavior (COM-B) model behavior change wheel to comprehensively explore factors influencing the acceptability of AI-based health technologies among older adults. The TDF consists of 14 domains that integrate behavioral determinants derived from over 30 psychological theories [18]. To ensure comprehensive coverage of these domains, the interview guide focused on five key areas: (1) general impressions of AI, (2) previous experiences with AI-based technologies, (3) perceptions and attitudes toward AI-based health technologies, (4) expected difficulties and underlying reasons, and (5) willingness. These focus areas were aligned with the study objectives and systematically mapped to the relevant TDF domains, as summarized in Table 1. This approach ensured that all 14 domains were addressed, either directly or indirectly, during the interviews.

**Table 1.** The interview questions guided by the Theoretical Domains Framework (TDF).

Focus area	Aligned TDF domains	Example question
General impressions of AI^a^	Knowledge, emotion, and optimism	“What comes to mind when you think about artificial intelligence and its role in health technologies?”
Previous experiences with AI-based technologies	Memory, attention, and decision processes; knowledge; and skills	“Have you used any AI-based technologies before? Can you describe your experience?”
Perceptions and attitudes toward AI-based health technologies	Social or professional role and identity; beliefs about capabilities; beliefs about consequences; emotion; and optimism	“What are your thoughts about using AI-based technologies for managing your health?”
Expected difficulties and underlying reasons	Environmental context and resources; skills; social influences; behavioral regulation; and memory, attention, and decision processes	“What challenges do you think you might face when using AI-based health technologies? Why do you think these occur?”
Willingness	Intentions; goals; beliefs about capabilities; and reinforcement	“Would you be willing to use AI-based health technologies? Why or why not?”

a AI: artificial intelligence.

To further enhance the applicability of findings, the identified TDF domains were mapped to the COM-B behavior change wheel (Table 2). The COM-B model summarizes 6 sources of behavior—social or physical opportunity, automatic or reflective motivation, and physical or psychological capability. This model provided a practical framework for identifying specific behavior change factors and strategies to improve the acceptance and adoption of AI-based health technologies among older adults [19].

**Table 2.** Capability, Opportunity, Motivation, and Behavior (COM-B) model components and its relation to Theoretical Domains Framework (TDF) domains.

COM-B components		TDF domains
**Capability**		
	Psychological capability	Knowledge Skills Memory, attention, and decision processes
	Physical capability	Behavioral regulation Skills
**Opportunity**		
	Social opportunity	Social influences
	Physical opportunity	Environmental context and resources
**Motivation**		
	Reflective motivation	Social or professional role and identity Beliefs about capabilities Optimism Beliefs about consequences Intentions Goals
	Automatic motivation	Social or professional role and identity Optimism Reinforcement Emotion

The interview guide included open-ended questions designed to elicit rich, detailed responses.

This structured mapping ensured that the interview guide covered all 14 TDF domains comprehensively, while tailoring the questions to elicit insights into factors influencing the acceptability of AI-based health technologies among older adults. While alternative models, such as the technology acceptance model and the unified theory of acceptance and use of technology are commonly used in technology adoption studies, these models primarily focus on individual perceptions, such as perceived usefulness and ease of use (technology acceptance model) or performance expectancy and effort expectancy (unified theory of acceptance and use of technology). These constructs, while valuable, may not fully capture the complex interplay of factors influencing older adults’ adoption of AI technologies.

The systematic alignment between the interview guide and the TDF framework facilitated the identification of theoretical mediators and behavioral determinants. This mapping subsequently informed the integration of the findings with the COM-B behavior change wheel, allowing for the development of tailored strategies to promote the use of AI-based health technologies in this population.

### Objectives

The objectives of the study were (1) to investigate the attitudes and perceptions of older adults toward the use of AI-based health technologies; (2) to identify potential facilitators, barriers, and challenges influencing older adults’ preferences toward AI-based health technologies; and (3) to inform strategies that can promote and facilitate the use of AI-based health technologies among older adults.

## Methods

### Study Design

A qualitative descriptive design was adopted for this study [20]. This approach helps in the effort to uncover and understand the experiences, attitudes, and perceptions of people, making it appropriate for this study. The findings from a qualitative descriptive study are able to inform strategies that promote and facilitate the use of AI-based health technologies, which makes it particularly useful for this research. To explore perceptions on and acceptability of the use of AI-based technologies in health maintenance among older adults, and to provide insights into their subjective views, in-depth semistructured interviews guided by an interview guide were conducted.

### Participants and Recruitment

Community-dwelling older adults aged 60 years or above were recruited from a local community center using a convenience sampling method. Eligible participants included those with or without previous exposure to AI tools who were willing to participate and share their experiences. Exclusion criteria included underlying physical, psychological, or neurodevelopmental problems that impaired interaction, mental instability, cognitive impairments such as traumatic brain injury, substance abuse, dementia, severe psychiatric disorders, intellectual disability, or being bedridden.

### Sampling and Sample Size

Convenience sampling approach was employed, guided by the principle of data saturation. The target sample size was 25 participants, which is commonly regarded as sufficient for qualitative descriptive studies to achieve thematic depth and richness [21]. Ultimately, 27 older adults were recruited, providing adequate data to explore the study’s focus areas.

### Data Collection

Data collection involved 3 sessions of semistructured interviews with each participant, each lasting 1 hour. To minimize ambiguity, AI was classified into 4 categories: machine learning, NLP, physical robots, and robotic process automation. Interviews covered five focus areas: (1) general impressions of AI; (2) previous experiences with AI-based technologies; (3) perceptions and attitudes toward AI-based health technologies; (4) anticipated difficulties in using AI-based health technologies and their underlying reasons; and (5) willingness, preferences, and motivations in accepting AI-based health technologies.

In the first focus area, examples of AI tools were not initially provided to elicit spontaneous responses. However, recognizing that participants might not be fully aware of everyday interactions with AI, the second focus area included real-life examples to clarify potential misconceptions. Examples of machine learning technologies included fitness tracking devices, such as Google Fitbit and Apple Watch, which monitor physical activity and provide insights into health trends. For NLP, the interviewer referenced virtual assistants like Siri (Apple Inc) and Alexa (Amazon Inc), as well as health care chatbots that answer patient inquiries. Computer vision was illustrated with examples of AI systems that analyze medical images, such as detecting abnormalities in x-rays or CT scans. This approach enabled a nuanced exploration of participants’ experiences with general AI technologies versus their attitudes toward AI for health purposes. The data were securely stored in Cloud storage, accessible only to the research team.

### Data Analysis

Thematic analysis was used in this study [22]. The interview recordings were first translated into English, transcribed, read, and reread to gain a sense of the participants’ experiences. Initial ideas were noted down. Next, the data were coded to extract key insights from the transcript. The codes were reviewed in a weekly discussion with the supervisor to obtain agreement on the interpretations. The underlying subthemes that emerged from the codes were identified and the subthemes were further clustered into themes. The themes, subthemes, and representative quotes were used to present the findings. Data were mapped to TDF to identify underlying theoretical mediators that influenced the perceptions and acceptability to the older adults of the use of AI-based health technologies. Once these theoretical domains were identified, they were mapped to components of the COM-B behavior change wheel that matched theoretical mediators with corresponding key strategies.

### Ethical Considerations

The study protocol and procedures were approved by the Ethical Committee of the Hong Kong Polytechnic University (approval HSEARS20230810006) on August 29, 2023, and adhered to the ethical standards of the Declaration of Helsinki. Before starting the study, all participants provided informed consent during a face-to-face interview with the researcher. All the participants were fully informed about the nature and purpose of the study. They were guaranteed the right to withdraw from the study at any time without adverse consequences. The collected data were encrypted and stored in a password-protected database. Although the researchers did not foresee any significant risks associated with the proposed study, the researchers recognized that older adults might experience discomfort or fatigue from sitting through the interviews. To address this, the researchers ensured that participants were given scheduled breaks throughout the interview. In addition, participants were encouraged to request breaks as needed to ensure that they were comfortable during the entire interview process. Participants who completed the interviews received an HK $100 (US $12.80) supermarket gift voucher to compensate for transportation costs.

## Results

### Participant Characteristics

The study included 27 participants, with 18 females (66.7%) and 9 males (33.3%). The mean age was 69.44 (SD 6.7) years. Most participants were married (19/27, 70.4%) and had a high school education (14/27, 51.9%). A majority lived with family (19/27, 70.4%) and rated their health as good (14/27, 58.3%). In addition, 66.7% (18/27) reported having chronic diseases. Regarding technology, 51.9% (14/27) felt somewhat comfortable using smartphones or tablets, and 59.3% (16/27) were somewhat familiar with tech products. Over 80% (23/27, 85.2%) of participants had heard of AI health technology, though their familiarity was limited. AI product usage varied, with approximately half of the participants reporting they used AI products often or occasionally, while the other half reported they used them seldom or were unfamiliar with them. Most participants (21/27, 77.8%) believed AI was to some extent helpful in health management, and 66.7% (18/27) had a positive overall impression of AI. Further details about the characteristics of the participants are provided in Table 3.

**Table 3.** Participant characteristics.

Characteristic		Total (N=27)	
**Gender, n (%)**			
	Men	9 (33.3)	
	Women	18 (66.7)	
**Age (y)**			
	Mean (SD)	69.44 (6.17)	
	Median (IQR)	70 (66-74)	
**Marital status, n (%)**			
	Single	1 (3.7)	
	Married	19 (70.4)	
	Divorced	1 (3.7)	
	Widowed	6 (22.2)	
**Education level, n (%)**			
	Primary school and below	4 (14.8)	
	Junior high school	7 (25.9)	
	High school	14 (51.9)	
	College or university	2 (7.4)	
	Postgraduate degree	0 (0)	
**Living status, n (%)**			
	Live with family	19 (70.4)	
	Live alone	7 (25.9)	
	Did not complete	1 (3.7)	
**Self-rated health status, n (%)**			
	Poor	1 (3.7)	
	Fair	12 (44.4)	
	Good	14 (58.3)	
**Any chronic diseases, n (%)**			
	Yes	18 (66.7)	
	No	9 (33.3)	
**Comfort level with using smartphone or tablet, n (%)**			
	Very comfortable	10 (37.0)	
	Somewhat comfortable	14 (51.9)	
	Not very comfortable	2 (7.4)	
	Completely uncomfortable	0 (0)	
	Did not complete	1 (3.7)	
**Familiarity with technology products, n (%)**			
	Very familiar	0 (0)	
	Somewhat familiar	16 (59.3)	
	Not very familiar	8 (29.6)	
	Completely unfamiliar	2 (7.4)	
	Did not complete	1 (3.7)	
**Have heard of AI**^**a**^ **health technology, n (%)**			
	Yes, very familiar	0 (0)	
	Yes, somewhat familiar	11 (40.7)	
	Yes, not very familiar	12 (44.4)	
	No, completely unfamiliar	3 (11.1)	
	Did not complete	1 (3.7)	
**Frequency of AI product usage, n (%)**			
	Often used	6 (22.2)	
	Occasionally used	6 (22.2)	
	Seldom used	5 (18.5)	
	Never used	7 (25.9)	
	Did not complete	3 (11.1)	
**Attitude toward AI’s helpfulness in health management, n (%)**			
	Very helpful	2 (7.4)	
	To some extent helpful	21 (77.8)	
	Not helpful	0 (0)	
	Uncertain	2 (7.4)	
	Did not complete	2 (7.4)	
**Overall impression about AI technology, n (%)**			
	Positive	18 (66.7)	
	Neutral	6 (22.2)	
	Negative	0 (0)	
	Uncertain	1 (3.7)	
	Did not complete	2 (7.4)	

a AI: artificial intelligence.

### Objective 1: Investigate Older Adults’ Attitudes and Perceptions Related to the Use of AI-Based Health Technologies

#### General Impressions of AI-Based Health Technologies

Most participants had positive views of AI-based health technologies, recognizing their potential to improve health outcomes by enhancing health monitoring, providing personalized recommendations, and assisting in decision-making. However, there was also limited awareness and understanding of specific AI-based health technologies, with some participants confusing them with general technologies such as smartphones. In addition, concerns were raised about fraud and scams associated with AI-based health technologies.

#### Attitudes and Perceptions Toward AI-Based Health Technologies

Privacy and data security were recurring themes in participants’ discussions of AI-based health technologies. Many participants expressed concerns about the potential misuse of personal data and highlighted the importance of robust security measures. These concerns were particularly pronounced among participants with limited experience using technology or those influenced by media reports of data breaches. However, a subset of participants viewed privacy as a societal issue rather than a specific risk associated with AI-based technologies. For instance, one participant remarked:

*Yes, it’s acceptable….Uh, what privacy do you have? There is no privacy in the whole world. Everyone’s mobile phone is being monitored*.

[Participant 3]

Another noted:

*Not to mention privacy nowadays, from where we sit now there is completely no privacy…even if you listen to this song now, then this song will automatically appear (on your phone). When you are viewing clothes, then you will see the clothes later on (on your phone*).

[Participant 5]

These contrasting perspectives highlight the varying levels of concern about privacy among older adults and underscore the need for transparent communication about data security in the development and deployment of AI-based health technologies.

#### Trustworthiness and Accuracy

Trust in AI-based health technologies varied among participants, influenced by previous experiences, information sources, and perceptions of accuracy. While some acknowledged the utility of AI in self-monitoring, others expressed reservations about its accuracy and trustworthiness, stressing the irreplaceable value of human judgment and expertise in health care.

### Objective 2: Identify Potential Facilitators, Barriers, and Challenges That Influence Older Adults’ Preferences Toward AI-Based Health Technologies

#### Technological Barriers

Participants identified multiple technological challenges that hindered their ability to use AI-based health technologies effectively. These challenges included operational difficulties, limited digital literacy, and lack of access to required resources, such as hardware or stable internet connectivity. These barriers were particularly evident among participants with physical or cognitive impairments due to age-related degeneration.

For instance, 1 participant described how physical limitations affected their interaction with technology:

*I have degeneration, too. I could remember a lot of things before. But now I can’t. My body movements are slow now, so it’s difficult for me to use AI-based health technologies*.

[Participant 3]

Another significant barrier was self-perceived incompetence in using digital tools, often leading to frustration or feelings of inadequacy:

*“I feel like I am stupid after learning something*....”

[Participant 7]

In addition, resource-related challenges, such as a lack of access to necessary hardware (eg, smartphones) and stable internet connections, were raised, further limiting participants’ ability to engage with AI technologies.

#### Emotional and Psychological Barriers

Emotional and psychological factors emerged as significant barriers to the adoption of AI-based health technologies. These included fear of technology, skepticism regarding its reliability, and concerns about losing human connection in health care interactions. The prevalence of scams and fraudulent activities in the digital age further compounded participants’ fears, making them hesitant to trust digital tools.

For example, 1 participant expressed a deep apprehension about interacting with technology:

*I don’t feel comfortable with these technologies because they seem complicated, and I worry about making mistakes.*

[Participant 6]

Concerns about scams were also prevalent, with another participant noting:

*Nowadays, you hear so much about fraud. It makes me scared to trust anything online, even if it looks helpful.*

[Participant 4]

These findings illustrate that emotional and psychological barriers are not only about individual fears but also reflect broader societal concerns regarding trust and safety in the digital world.

#### Perceived Usefulness and Relevance

Participants perceived AI-based health technologies as useful, particularly those that addressed specific health needs such as the monitoring of blood pressure, blood sugar, and cholesterol. They also appreciated technologies that could assist with health monitoring, reminders, and personalized care.

*I hope that these three things could be more mature, help to reduce blood pressure, blood sugar, or blood cholesterol, whatever. At least I know whether my blood sugar is good or not; if it is not good, I will eat something and exercise to increase my physical capacity*.

[Participant 3]

*Yeah, like an alarm. As a reminder to alert you. Well, high blood pressure, high blood sugar, high cholesterol level, those we get when are older. If AI can help with these three things, it is definitely good*.

[Participant 2]

#### Acknowledgment by Authorities

The endorsement of AI-based health technologies by official authorities or regulatory bodies significantly facilitated their acceptance and adoption. Participants felt more confident and trusting when these technologies were validated by reputable sources, such as health care agencies or government bodies.

*If it is something organized by the Hospital Authority or the government, it will be more trustworthy. Because sometimes, if it is made by pharmaceutical companies, it may not necessarily be that clear*.

[Participant 2]

*Well, if you talk about it being official, like the doctors and government, I will assume that this AI is reliable, you will basically feel at ease*.

[Participant 1]

*I have to see if the AI comes from a large or a small company. For example, Hong Kong Polytechnic University, Baptist University — they are more reliable then.*

[Participant 4]

### Objective 3: Inform Strategies That Promote and Facilitate the Use of AI-Based Health Technologies Among Older Adults

#### Integration of Human Expertise

While recognizing the potential benefits of AI, participants emphasized the need for AI technologies to complement rather than replace human expertise in health care. They suggested that AI could be useful as an auxiliary tool for making preliminary medical suggestions, but that the final decision-making should involve consultations with a human being.

*I just ask the AI, but I can ignore its answers or I will think about it again. It is just a reference and I won’t believe it completely. Well, when you see a doctor, you won’t just see one doctor, you will see many many right?...AI is just the first contact point*.

[Participant 2]

#### User-Friendly Design

To enhance the acceptability of AI-based health technologies, it is crucial to develop solutions that are user-friendly and tailored to the specific needs of older adults. A user-friendly design should include simplified and intuitive interfaces that minimize cognitive load, with features such as large, high-contrast text and icons for better visibility and ease of use. Voice command functionality can further improve accessibility for users with limited dexterity or vision impairments. In addition, providing step-by-step tutorials, user manuals in multiple formats (eg, videos and print), and accessible customer support can address challenges related to digital literacy. These features collectively ensure that the technology is not only easy to use but also aligns with the physical and cognitive capabilities of older adults, thereby fostering greater adoption and satisfaction.

#### Addressing Privacy and Security Concerns

Given the concerns around privacy and data security, the development of AI-based health technologies should include robust security measures to protect the personal information of users. Addressing these concerns is essential to building trust and encouraging adoption among older adults.

#### Government and Regulatory Support

Participants highlighted the importance of government and regulatory support in promoting the ethical implementation of AI in health care. Clear guidelines and endorsements from authoritative bodies can foster trust and enhance the acceptability of AI-based health technologies among older adults.

Table 4 provides a detailed description of how the identified barriers and facilitators toward the use of AI-based health technologies were mapped to the TDF domains and corresponding COM-B components. These mappings were derived from participants’ responses during the interviews. The barriers and facilitators were categorized based on their alignment with the TDF domains and subsequently mapped to the COM-B components to identify actionable strategies for behavior change.

**Table 4.** Identified Theoretical Domains Framework (TDF) domains and Capability, Opportunity, Motivation, and Behavior (COM-B) components related to the adoption of artificial intelligence (AI)–based health technologies.

Barrier or facilitator	COM-B components	TDF domains	Illustrative participant quote
Barrier: limited technological skills	Capability	Knowledge and skills	“I find it difficult to use these technologies because my body movements are slow, and I don’t know how to start.” [P3]
Facilitator: perceived usefulness and relevance	Motivation	Beliefs about consequences, intentions, and goals	“If AI can help monitor my blood pressure or remind me, it would be very helpful.” [P2]
Barrier: lack of official recognition or endorsement	Opportunity	Social influences	“If it’s recognized by the government or hospitals, I would trust it more.” [P1]
Facilitator: confidence in trustworthiness and accuracy	Motivation	Beliefs about capabilities	“If the results are accurate, I can rely on it for some guidance.” [P4]
Barrier: privacy and data security concerns	Motivation	Emotion	“I worry about my personal information being stolen if I use this technology.” [P5]
Facilitator: AI as an auxiliary tool for initial suggestions	Motivation	Beliefs about consequences and intentions	“I can use AI for some advice, but I still prefer to consult a doctor for the final decision.” [P2]

## Discussion

### Principal Findings

The aim of the study was to provide insights into the perceptions and acceptability of AI-based health technologies among older adults, using the TDF and the COM-B behavior change wheel as theoretical frameworks. These frameworks were instrumental in identifying key behavioral determinants and mapping them to actionable strategies for enhancing AI adoption. In total, 9 of the 14 TDF domains (knowledge, skills, social influences, environmental context and resources, beliefs about capabilities, beliefs about consequences, intentions, goals, and emotion) were identified and mapped to 6 COM-B components (psychological capability, physical capability, social opportunity, physical opportunity, reflective motivation, and automatic motivation). By applying these frameworks, the study provided a structured approach to understanding the factors influencing older adults’ behavior toward AI adoption. The responses of the participants shed light on various aspects of the adoption and utilization of AI-based health technologies among older adults. First, the participants expressed a range of attitudes and perceptions toward AI-based health technologies, including curiosity, skepticism, and enthusiasm toward the use of AI in the management and maintenance of health. These perceptions align with the TDF domains of knowledge and beliefs about consequences, which underscore the need to enhance understanding of AI and clearly communicate its benefits to older adults. The interviews provided a deeper understanding of the factors influencing these attitudes and the potential benefits and concerns associated with AI adoption in the context of the participants’ health. They shared their perceptions of AI technologies, addressing aspects such as reliability, trustworthiness, and their perception of the impacts on health care outcomes. These insights informed the reflective motivation component of the COM-B framework, highlighting the importance of fostering trust and reliability in AI-based technologies to enhance adoption.

In addition, the study revealed an interesting finding regarding older adults’ preferences for using AI in asking for advice and self-health maintenance. Contrary to expectations, participants expressed a higher level of trust and reliability in real persons, particularly physicians, when making decisions about pharmacological interventions such as changing drug dosages. Participants believed that AI could play a valuable role in providing information and acting as a reference tool for self-health maintenance. However, they expressed reservations about relying solely on AI for decisions related to medication management. This finding is linked to the TDF domain of social influences and the social opportunity component of the COM-B framework, as participants emphasized the irreplaceable role of human expertise and interpersonal trust in health care decision-making. They viewed AI as a useful source of information but considered the expertise and experience of physicians and other health care professionals to be more reliable and crucial in making decisions regarding medical interventions and treatment. The emotion domain of TDF also played a role, as participants’ skepticism and emotional barriers highlighted the importance of addressing concerns through education and reassurance. The findings were also in line with a mixed methods study indicating that the lack of empathy and a professional human approach made AI chatbots less acceptable to some users [15]. This insight has significant implications for the development of AI-based health technologies. It might indicate that older adults desire a collaborative approach that combines the benefits of AI with the guidance and expertise of real-person health care providers [23-25]. Developers could focus on creating AI systems that could provide accurate and evidence-based information, acting as a reference tool to support older adults’ self-health maintenance efforts. In this context, AI could assist older adults in accessing reliable and up-to-date information about medications, potential side effects, and alternative treatment options. AI-based systems could also offer personalized recommendations for lifestyle modifications, nonpharmacological interventions, and self-care strategies that align with the preferences and needs of older adults.

While privacy and confidentiality are often considered to be potential concerns when using AI in health care, surprisingly, many expressed a relaxed and relatively pessimistic attitude toward privacy. One of the reasons for this was that the perceived benefits outweighed the concerns. Most expressed the view that the potential benefits of AI-based health technologies have overshadowed any concerns about privacy or confidentiality. They may have viewed the potential improvement in their health outcomes or access to personalized care as more significant than the potential risks to their privacy. Another underlying reason may be that the participants may have had other concerns that they considered more important than privacy and confidentiality in the context of AI-based health technologies. For example, they may have been more focused on the usability, effectiveness, or reliability of the technology. Regardless, the absence of expressed concerns does not necessarily indicate the lack of importance of privacy and confidentiality. Rather, it highlights the need for researchers and developers to proactively address these concerns and ensure that robust privacy and security measures are in place when designing and implementing AI-based health technologies. It is crucial for future research and development efforts to emphasize the importance of privacy and confidentiality in AI-based health technologies, which involves implementing strong data protection measures, obtaining informed consent from users, and transparently communicating how their data will be used and safeguarded.

To enhance older adults’ acceptance of AI-based health technologies, strategies should focus on improving technological skills through tailored training programs, securing official recognition and endorsement from health care authorities, addressing concerns over privacy and data security with robust protocols, emphasizing the trustworthiness and accuracy of AI tools, positioning AI as auxiliary aids for medical suggestions, highlighting the usefulness and relevance of AI applications, and providing emotional support to mitigate psychological barriers. These strategies are directly informed by the COM-B framework, targeting capability, opportunity, and motivation as the key drivers of behavior change. By implementing these targeted strategies, older adults might be encouraged to embrace AI technologies, leading to improved health care outcomes and increased engagement with innovative health care solutions. Meanwhile, government endorsements could also contribute to the establishment of guidelines, standards, and regulations that ensure the ethical use of AI in health care, addressing concerns related to privacy, data security, and quality assurance.

### Limitations

The limitations of this study were the sample size and representativeness of participants. While we recognize that this sample size may not fully capture the diversity of the population, the study aimed to offer initial, in-depth insights into older adults’ perceptions of AI-based health technologies. Future studies could address subgroup comparisons by incorporating stratified sampling to examine variations across different demographic or socioeconomic groups. In addition, while quantitative comparisons were not a focus of this study, future research could include mixed method approaches to statistically assess participant perceptions and examine their relationships with demographic and contextual factors by using validated survey tools. Last but not the least, many participants had limited before knowledge or experience with AI-driven health technologies. Some participants conflated AI technologies with general digital tools, such as mobile apps, due to a lack of familiarity with the distinction. To address this, the interviewer provided concise explanations and real-life examples of AI subsets, including machine learning, NLP, computer vision, and expert systems. While this approach helped participants better understand the discussion, their responses were still shaped by their interpretations and familiarity with technology. Consequently, the findings reflect older adults’ perceptions and acceptance of health technologies they associate with AI, rather than definitive perspectives on AI-driven health technologies.

### Conclusion

While the majority of participants recognized the potential advantages of AI-based health technologies, they underscored the indispensable role of human expertise and interaction. Vital strategies for improving acceptability include crafting user-friendly and customized AI solutions, addressing privacy concerns, ensuring robust security measures, and integrating AI as a complementary tool alongside health care providers. Government backing and guidelines can play a pivotal role in advancing ethical AI integration in health care, fostering trust, and enhancing personalized care for older adults, ultimately leading to improved health outcomes in this population.

## Abbreviations

AI: artificial intelligence
COM-B: Capability, Opportunity, Motivation, and Behavior
NLP: natural language processing
TDF: Theoretical Domains Framework
